# The Correlation Between Falls and Cognitive Frailty in Elderly Individuals With Hypertension in a Chinese Community

**DOI:** 10.3389/fnagi.2022.783461

**Published:** 2022-05-10

**Authors:** Can Wang, Yue Chong, Ling Wang, Yanbo Wang

**Affiliations:** ^1^Clinical Research Center for Mental Disorders, Shanghai Pudong New Area Mental Health Center, School of Medicine, Tongji University, Shanghai, China; ^2^Division of Medical Humanities and Behavioral Sciences, School of Medicine, Tongji University, Shanghai, China

**Keywords:** cognitive frailty, fall, aged, hypertension, mild cognitive impairment, physical frailty

## Abstract

**Background:**

Cognitive frailty refers to the presence of both physical frailty and mild cognitive impairment without simultaneous diagnosis of Alzheimer's disease or other dementia. Epidemiological studies have confirmed the correlation between falls and cognitive frailty, but no study has investigated the relationship between fall risk and cognitive frailty in hypertensive elderly Chinese individuals.

**Methods:**

From December 2020 to March 2021, during face-to-face interviews, community-dwelling elderly individuals with hypertension aged 60~89 in Pudong New Area, Shanghai, were evaluated for cognitive frailty, fall history, and depression, and sociodemographic characteristics were collected. Logistic regression was used to analyze the correlation between falls and cognitive frailty.

**Results:**

A total of 305 elderly people were investigated in this study, and 173 (56.7%, 95% CI =51.2%~62.2%) reported falling once or more in the previous year. Cognitive frailty is closely related to falls and was an independent risk factor for falls (OR = 2.661, 95% CI = 1.063~6.659). Other risk factors included old age (OR = 4.306, 95% CI = 1.852~10.013), female sex (OR = 1.988, 95% CI = 1.185~3.335) and depression (OR = 2.936, 95% CI = 1.069~8.060).

**Conclusion:**

Cognitive frailty is an important risk factor for falls in elderly individuals with hypertension in Chinese communities.

## Introduction

The World Health Organization defines a fall as “an event that causes a person to inadvertently rest on the ground, floor or other lower level” (World Health Organization, 2008). In China, the leading cause of injury-related death in adults over 60 years old is falling (Robertson et al., 2013). Every year, one-third of adults over 65 years old and half of adults over 80 years old fall (6). Falls and fall-related injuries have become the major health problem among elderly individuals. Scholars have conducted extensive research on the risk factors for falls, including sex, age, muscle weakness, gait and balance disorders, visual impairment, foot or ankle diseases, history of falls, fear of falls, polypharmacy and so on (Sharif et al., 2018). Early identification of risk factors associated with falls can help identify individuals who may benefit from appropriate interventions and prevent future injuries (Guirguis-Blake et al., 2018).

As of 2020, there are 264 million people over 60 in China, accounting for 18.7% of the total population. Population aging leads to a significant increase in age-related diseases, such as frailty and cognitive decline. Because they have a common pathophysiological mechanism (Gallucci et al., 2018; Aguilar-Navarro et al., 2019; Bortone et al., 2021), frailty and cognitive decline interaction accelerates declines in the physical and cognitive function of elderly individuals. Therefore, the concept of cognitive frailty (CF) has been proposed and attracted much attention. Cognitive frailty refers to the presence of both physical frailty (PF) and mild cognitive impairment (MCI) without simultaneous diagnosis of Alzheimer's disease or other dementia (Panza et al., 2018). Studies have found that cognitive impairment and PF increase the risk of falls (Quach et al., 2019; Chittrakul et al., 2020; Hu et al., 2021; Song et al., 2021), and CF can better predict falls and other adverse consequences, such as disability, dysfunction and death, in an aging population (Aprahamian et al., 2018; Hao et al., 2018; Panza et al., 2018; Sugimoto et al., 2018; Bu et al., 2021; Zhang et al., 2021). Previous studies have shown that among elderly people living in urban and rural communities in China, CF is independently related to falls (Ma et al., 2017) (OR = 6.653, 95% CI = 2.651~16.697).

Although the abovementioned correlation between CF and falls has been reported, no similar study has been carried out in the hypertensive elderly population. We have learned that hypertension, cognitive impairment and frailty are common in elderly individuals, that the incidence of all of these conditions increases with age (Jing et al., 2019), and that hypertension is a common risk factor for PF and cognitive decline (Fougère et al., 2017; Ungvari et al., 2021). Therefore, we believe it is necessary to focus our research perspective on a specific group of hypertensive elderly. We aimed to explore whether the occurrence of falls in this group of elderly is associated with CF and whether this association is stronger than the association between falls and CF in elderly people without a diagnosis of hypertension. The 2019 edition of the Chinese guidelines for the management of hypertension in the elderly also emphasizes the importance of assessing CF and adverse outcomes in hypertensive elderly people (Li et al., 2019). Our previous study (Wang et al., 2021) investigated the prevalence and influencing factors of CF in elderly individuals with hypertension in China. On the basis of that study, we continued to explore the correlation of CF with falls. As far as we know, this is the first time this relationship has been examined in China. The purpose of this study was to understand the correlation between falls and CF in elderly individuals with hypertension in China and to provide a basis for understanding fall prevention and CF in elderly individuals with hypertension.

## Materials and Methods

### Research Design and Subjects

**Figure 1:**
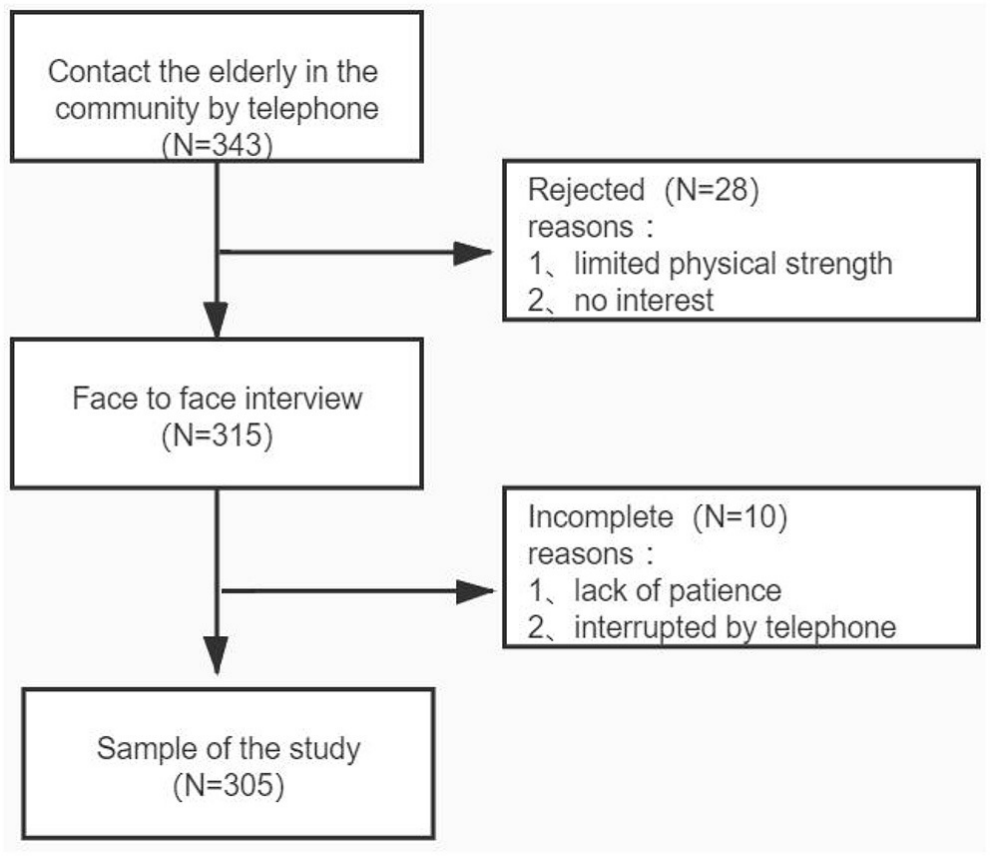
Participant screening flowchart.

This cross-sectional study was conducted in the Pudong New Area of Shanghai from 1 December 2020 to 31 March 2021. With the assistance of neighborhood committee staff, we recruited participants from the older adult population living in the community who met the following inclusion criteria by telephone: (1) 60~89 years old; (2) received antihypertensive treatment or had a history of hypertension. Subjects with dementia and serious mental disorders (such as schizophrenia, bipolar disorder, etc.) were excluded. We contacted a total of 343 older adults, of whom 28 refused to participate in the study because of “limited physical strength” and “no interest.” Ten did not complete the test because of lack of patience or being interrupted during the telephone interview. Finally, 305 older adults were included in the study. The research protocol was approved by the Ethics Committee of Pudong New Area Mental Health Center (PDJWMLL2020025). We fully explained the research process to all participants and obtained written informed consent. The study flowchart is shown in Figure 1.

### Falls

Falls were evaluated by the question “Have you fallen in the past 12 months?” Participants were divided into two groups: (1) no falls and (2) one or more falls.

### Outcomes

The primary outcome of our study was the correlation between falls and cognitive frailty in elderly individuals with hypertension, and the secondary outcome was the incidence of falls and other influencing factors.

### Sociodemographic Characteristics

The sociodemographic characteristics collected included sex, age, duration of hypertension, body mass index (BMI), degree of education, and monthly salary. The age groups were 60~69 years old, 70~79 years old and 80~89 years old. The duration of hypertension was divided into 1~5 years, 6~10 years and > 10 years. The diagnosis and duration of hypertension were collected from patient self-reports and medical insurance records. BMI was divided into underweight (<18.5 kg/m^2^), normal weight (18.5~24.9 kg/m^2^), and overweight and above (≥ 25.0 kg/m^2^). Education level was divided into four categories: illiterate, primary school, junior middle school, and senior high school and above. Monthly salary was divided into three ranges: <1000 yuan, 1,000~3,000 yuan and more than 3,000 yuan.

### Cognitive Frailty

CF refers to PF and MCI (excluding dementia) in older adult individuals. We used the Mini Mental State Examination (MMSE) (Folstein et al., 1975) and Frailty Phenotype (FP) (Fried et al., 2001) to measure CF. The MMSE (Folstein et al., 1975) is used to evaluate the cognitive function of individuals. The score ranges between 0 and 30. The higher the score is, the better the cognitive function. The scores used to diagnose MCI were as follows: for ≤ 75 years old with elementary school education and below, the MMSE score is 23.5–26.5, and above elementary school is 23.5–28.5; for >75 years old with elementary school education and below, the MMSE score is 19.5–22.5, and above elementary school is 23.5–26.5.The frailty phenotype (Fried et al., 2001) is used to evaluate PF and includes five physiological indicators: involuntary weight loss, fatigue, reduction in grip strength, reduction in walking speed and reduction in physical activity. The total score ranges from 0 to 5. A score of 0 indicates no frailty, a score of 1 or 2 indicates the prophase of frailty, and a score from 3 to 5 indicates frailty. We have to admit that our coarse diagnosis of MCI is one of the main limitations of this study, because we only used MMSE to diagnose MCI and did not assess independence in activities of daily living.

### Depression

The Geriatric Depression Scale (GDS-15) was used to evaluate the depression status of the older adult participants. The GDS-15 is a simplified version of the GDS and was revised in 1986 (Cruice et al., 2011). There are 15 items in total. Each item is scored as 1 or 0 according to whether the response is “yes” or “no,” respectively, and negative questions are scored in reverse. The total score ranges from 0 to 15. The higher the score is, the higher the level of depression. A score from 0~4 indicates no depression, 5~7 indicates mild depression, 8~11 indicates moderate depression, and 12~15 indicates severe depression. The sensitivity and specificity of the GDS-15 were 0.96 and 0.88, respectively. The α coefficient was 0.82.

### Data Analysis

Excel was used to establish the database with double data entry, and SPSS 26.0 (Windows version, IBM) was used to describe and analyze the data. The Kolmogorov-Smirnov test was used to test the normality of the distributions of continuous variables. The measurement data conformed to normal distributions, and means ± standard deviations are used to describe these variables. The count data are expressed as frequencies (percentages). The chi-square test was used to determine the differences between groups in terms of demographic and clinical characteristics. Binary logistic regression (LR) was used to analyze the correlation between falls and cognitive impairment in elderly individuals with hypertension, and the backward LR method was used to screen out statistically significant variables. All statistical tests were two-sided, with *p* < 0.05 indicating statistical significance.

## Results

A total of 173 of 305 elderly patients with hypertension fell in the last 12 months, and the prevalence of falls was 56.7% (95 CI% = 51.2%~62.2%). Other demographic data (sex, age, duration of hypertension, education level, BMI and monthly salary) and data on CF and depression are shown in Table 1. The results of the univariate analysis of falls in elderly patients with hypertension are as follows (see Table 1): there was no significant difference in the incidence of falls among elderly patients with hypertension by educational level, BMI or monthly salary (*P* > 0.05). There were significant differences in the incidence of falls among elderly hypertensive patients by sex, age, duration of hypertension, CF status, PF status and depression status (*P* < 0.05).

**Table 1:** General demographic data and univariate analyses.

**Variable**	**Number**	**Non-Fall (*n* = 132, 43.3%)**	**Fall (*n* = 173, 56.7%)**	**Statistical values**	***P*-values**
Sex				6.146	0.013
Male	108	57(43.2%)	51(29.5%)		
Female	197	75(56.8%)	122(70.5%)		
CF				8.045	0.005
No	268	124 (93.9%)	144 (83.2%)		
Yes	37	8 (6.1%)	29 (16.8%)		
MMSE		23.86 ± 2.176	23.97 ± 2.474	0.424	0.672
PF		1.24 ± 0.857	1.62 ± 1.097	3.254	0.001
Age group				22.492	0.000 <0.001
60–69	140	61 (46.2%)	79 (45.7%)		
70–79	114	63 (47.7%)	51 (29.5%)		
80–89	51	8 (6.1%)	43 (24.9%)		
Depression				8.762	0.003
No	273	126 (95.5%)	147 (85.0%)		
Yes	32	6 (4.5%)	26 (15.0%)		
Duration^※^				8.455	0.015
≤ 5 years	123	57 (43.2%)	66 (38.2%)		
6–10 years	82	43 (32.6%)	39 (22.5%)		
>10 years	100	32 (24.2%)	68 (39.3%)		
Education level				5.876	0.118
Illiterate	110	54 (40.9%)	56 (32.4%)		
Primary school	122	55 (41.7%)	67 (38.7%)		
Junior middle school	49	16 (12.1%)	33 (19.1%)		
Senior high school and above	24	7 (5.3%)	17 (9.8%)		
BMI				3.236	0.198
<18.5 kg/m^2^	130	59 (44.7%)	71 (41.0%)		
18.5–24.9 kg/m^2^	29	8 (6.1%)	21 (12.1%)		
>25 kg/m^2^	146	65 (49.2%)	81 (46.8%)		
Monthly salary				0.723	0.697
<1,000yuan	101	42 (31.8%)	59 (34.1%)		
1,000–3,000 yuan	128	59 (44.7%)	69 (39.9%)		
>3,000yuan	76	31 (23.5%)	45 (26.0%)		

*Duration^※^ refer to number of years with hypertension*.

Taking the occurrence of falls as the dependent variable and the statistically significant influencing factors from the univariate analysis (sex, age, duration of hypertension, CF status, PF status and depression status) as the independent variables, backward LR analysis was carried out. The inclusion standard was 0.05, and the exclusion standard was 0.10. The results (see Table 2) show that the risk of falls in female hypertensive elderly individuals is 1.988 times higher than that in males (95% CI = 1.185~3.335). The risk of falls in elderly individuals with CF was 2.661 times higher than that in elderly individuals without CF (95% CI = 1.063~6.659). Compared with elderly individuals aged 60~69, participants aged 80~89 had a 4.306-fold increase in risk of falling (95% CI = 1.852~10.013). The fall risk of depressed participants was 2.936 times higher than that of nondepressed participants (95% CI = 1.069~8.060).

**Table 2:** Backward selection logistic regression (LR) analysis of falls in elderly patients with hypertension.

**Variable**	**B**	**S.E**.	**Wald's**	**OR (95%CI)**	***P***
Sex					
Male				1	
Female	0.687	0.264	6.775	1.988 (1.185–3.335)	0.009
CF					
No				1	
Yes	0.979	0.468	4.372	2.661 (1.063–6.659)	0.037
Age group					
60–69				1	
70–79	−0.503	0.268	3.529	0.605 (0.358–1.022)	0.06
80–89	1.46	0.431	11.502	4.306 (1.852–10.013)	0.001
Depression					
No				1	
Yes	1.077	0.515	4.369	2.936 (1.069–8.06)	0.037

## Discussion

This study explored the relationship between falls and CF in elderly individuals with hypertension in China and factors influencing falling. Studies have found that CF is related to falls. However, no study has been carried out in elderly individuals with hypertension. To the best of our knowledge, this is the first study to examine the correlation between falls and CF in elderly individuals with hypertension in China. Our results show that there is a significant correlation between falls and CF in elderly individuals with hypertension.

In our study, the prevalence of falls in the elderly population was 56.7%, higher than the results of several other studies (13.1~25.4%) (Tsutsumimoto et al., 2018; Kim et al., 2019; Brigola et al., 2020; Zhao et al., 2020), which may be related to the fact that this study was carried out in an elderly population with hypertension. A meta-analysis (Shuqi et al., 2020) that included 11,493 hypertensive older adults revealed that history of falls, fear of falling, taking anti-hypertensive drugs and low medication adherence were all reasons for the high prevalence of falls in this population. In addition. These patients have rigid vessel walls, reduced pressure receptor sensitivity and they are more susceptible to emotions, infection, fatigue and other factors that eventually lead to falls. In the study by Ma et al., the fall rate of the elderly with hypertension was 77.8%, which was higher than that in our study, which may be related to the higher average age (75.2 ± 3.7) of the elderly in that study (Ma et al., 2021).

In this study, CF was an independent risk factor for falls in elderly individuals with hypertension. The risk of falls in elderly individuals with CF was 2.661 times higher than that in individuals without CF (OR = 2.661, 95% CI = 1.063~6.659). This suggests that CF needs to be closely evaluated in the context of fall prevention in elderly individuals with hypertension. A cross-sectional study (Zhao et al., 2020) in Japan divided 1,192 elderly people over 70 years old into three groups: the MCI group, frailty group and CF group. By asking about the fall in the previous year, it was finally found that the number of falls in the CF group (n = 12, 48%) was significantly higher than that in the other two groups (*P* = 0.005 <0.05), which was consistent with the results of Ma et al. (2017). In the study of Ma et al. (2021), considering the contingency of a single fall, the outcome variable of falling was defined as having had two or more falls. The participants were divided into two groups: (1) no falls or one fall and (2) 2 or more falls. The results showed that two or more falls were not related to non-CF status but were closely related to the presence of CF (OR = 3.51, 95% CI = 1.18~10.44). After adjusting for sociodemographic characteristics, lifestyle habits, chronic diseases and other factors, the difference was still significant (OR = 3.41, 95% CI = 1.11~10.50). Tsutsumimoto et al. (2018) collected data from 10,202 community-dwelling elderly individuals and founded that CF was associated with not only falls but also fall-related fractures. CF had a greater risk for fall-related fractures (OR = 1.92, 95% CI = 1.20~3.08, *P* = 0.007) than cognitive decline or physical frailty alone. Cadore et al. (2019) suggested that muscle strength, power output and weight loss are typical physiological characteristics of frailty, which leads to poor gait quality. Frailty is significantly associated with the risk of future falls (Cheng and Chang, 2017; Pérez-Ros et al., 2020; Rivera-Chávez et al., 2021). A study investigating the relationship between MCI and falls reported that the risk of falls is significantly higher in people with MCI (Quach et al., 2019) because declines in cognitive function reduce elderly people's access to and processing of external information, speed and execution. In addition, elderly individuals with cognitive impairment may act and react slowly and have visual and hearing impairments. When the environment changes unexpectedly, they cannot respond effectively and in a timely manner and easily fall. Of course, we also need to point out that the diagnosis of MCI in this study was made by the MMSE and did not combine the use of the Activities of Daily Living Scale (ADL) and the Clinical Dementia Rating Scale (CDR). The coarse diagnosis of MCI directly influenced the diagnosis of CF, which may have some influence on the conclusion of this study.

In the sociodemographic data of this study, the factors closely related to falls in the elderly population were old age (80~89 years old, OR = 4.306, 95% CI = 1.852~10.013) and female sex (OR = 1.988, 95% CI = 1.185~3.335), which is consistent with the results of several other studies (Kim et al., 2019; Brigola et al., 2020; Pirrie et al., 2020; Susilowati et al., 2020; Ma et al., 2021) in which the subjects were elderly in general (i.e., not distinguish whether or not they were diagnosed with hypertension). Aging is accompanied by different rates of degradation in human organs, tissues, muscles, cells and other components. Elderly individuals, especially very elderly individuals, have a high incidence of falls. In addition, the estrogen level of elderly women decreases, and the activity of osteoblasts and osteoclasts decreases, resulting in a significant decrease in bone mineral density, poor physical function and an increase in the prevalence of osteoporosis. Therefore, elderly women are more likely to fall.

We found that elderly individuals with depression had a higher risk of falling than those without depression (OR = 2.936, 95% CI = 1.069~8.060), which is consistent with the results of several other studies (Ouyang and Sun, 2018; Britton et al., 2019; Ma et al., 2021). In elderly individuals, there is a strong correlation between hypertension and depression, which interact with each other (Boima et al., 2020). Decreased physical activity in depressed elderly individuals causes an increase in inflammatory cytokines such as tumor necrosis factor (TNF) and interleukin (IL), further affecting skeletal muscle, reducing muscle density and accelerating the occurrence of osteoporosis (Ruan et al., 2017). In addition, depressed people often experience concurrent malnutrition (Alam et al., 2021) because under the influence of negative emotions, elderly individuals experience a decreased appetite, insufficient nutrition and energy intake, an irregular diet and gastrointestinal dysfunction. Malnutrition aggravates the age-related decline in muscle mass and strength (Sim et al., 2018; Viggiano et al., 2020). At the same time, due to the lack of nutrients such as serum protein, it also has an adverse impact on bone density, resulting in a significant increase in the risk of falls in elderly individuals (Sim et al., 2018). Some studies have also indicated that depression reduces attention to the external environment, and this decline in executive ability affects the ability to fully process information while completing dual tasks, such as walking and speaking, and further induces gait and balance problems, resulting in falls (Hoffman et al., 2017).

### Limitations

First, this study is a cross-sectional trial, and the exact relationship between falls and CF cannot be confirmed. We plan to conduct further follow-up tests on this cohort and examine longitudinal data in the future. Second, the use of self-reported data to measure falls may be biased. Third, there is no information about the falls that occurred, such as when the falls occurred and the activities being carried out at that time. Finally, the coarse diagnosis of MCI is one of the limitations of this study. We used only the MMSE to diagnose MCI and did not assess independence in activities of daily living. A more extensive neuropsychological assessment may help in the diagnosis of MCI.

## Conclusion

In summary, our results show that CF plays an important role in the fall risk of hypertensive elderly individuals in the Chinese community; older age (80~89 years old), female sex and depression were independent risk factors for falls. The results of this study are helpful for guiding fall prevention decisions.

## Data Availability Statement

The original contributions presented in the study are included in the article/supplementary material, further inquiries can be directed to the corresponding author.

## Author Contributions

CW collected data, drafted the initial manuscript, and reviewed and revised the manuscript. YC collected and imputed data, and drafted the initial manuscript. YW and LW conceptualized and designed the study, analyzed data, critically reviewed the manuscript for important intellectual content, and reviewed and revised the manuscript. All authors have read and agreed to the published version of the manuscript.

## Funding

This research was funded by Science and Technology Development Fund of Shanghai Pudong New Area (No. PKJ2020-Y31), Public Health Characteristic Discipline of Pudong New Area Health Commission (Grant No. PWYgts2021-01) and Medical Discipline Construction Project of Pudong Health Committee of Shanghai (Grant No. PWYgy2021-02).

## Conflict of Interest

The authors declare that the research was conducted in the absence of any commercial or financial relationships that could be construed as a potential conflict of interest.

## Publisher's Note

All claims expressed in this article are solely those of the authors and do not necessarily represent those of their affiliated organizations, or those of the publisher, the editors and the reviewers. Any product that may be evaluated in this article, or claim that may be made by its manufacturer, is not guaranteed or endorsed by the publisher.
